# Human stem cell transplantation models of Alzheimer’s disease

**DOI:** 10.3389/fnagi.2024.1354164

**Published:** 2024-02-21

**Authors:** Nkechime Ifediora, Peter Canoll, Gunnar Hargus

**Affiliations:** ^1^Department of Pathology and Cell Biology, Columbia University, New York, NY, United States; ^2^Taub Institute for Research on Alzheimer’s Disease and the Aging Brain, Columbia University, New York, NY, United States

**Keywords:** Alzheimer’s disease, induced pluripotent stem cells, IPSC, disease modeling, transplantation

## Abstract

Alzheimer’s disease (AD) is the most frequent form of dementia. It is characterized by pronounced neuronal degeneration with formation of neurofibrillary tangles and deposition of amyloid β throughout the central nervous system. Animal models have provided important insights into the pathogenesis of AD and they have shown that different brain cell types including neurons, astrocytes and microglia have important functions in the pathogenesis of AD. However, there are difficulties in translating promising therapeutic observations in mice into clinical application in patients. Alternative models using human cells such as human induced pluripotent stem cells (iPSCs) may provide significant advantages, since they have successfully been used to model disease mechanisms in neurons and in glial cells in neurodegenerative diseases *in vitro* and *in vivo*. In this review, we summarize recent studies that describe the transplantation of human iPSC-derived neurons, astrocytes and microglial cells into the forebrain of mice to generate chimeric transplantation models of AD. We also discuss opportunities, challenges and limitations in using differentiated human iPSCs for *in vivo* disease modeling and their application for biomedical research.

## Introduction

Alzheimer’s disease (AD) is one of the most prevalent neurodegenerative diseases and the most common form of dementia (Winblad et al., 2016). AD leads to slowly progressive cognitive decline with impairment of memory, learning and language and features of the pathology include deposition of amyloid β (Aβ) plaques in the neuropil, neurofibrillary tangles within neurons, synaptic and neuronal loss as well as neuroinflammation marked by reactive astrocytes and microglia in areas of neurodegeneration (Serrano-Pozo et al., 2011). White matter abnormalities have also been described in AD patient brains (Nasrabady et al., 2018).

Despite recent progress in clinical trials using monoclonal antibodies to reduce the burden of Aβ plaques in patients’ brains (Rabinovici and La Joie, 2023; Reardon, 2023; van Dyck et al., 2023), there is currently no cure for AD. Thus, there is a need to develop model systems to study disease mechanisms and to establish curative therapeutic strategies. Animal models of AD overexpressing familial AD (fAD) mutations in *APP* and/or *PSEN* demonstrate pronounced deposition of Aβ plaques as well as synaptic dysfunction and neuroinflammation, but they lack formation of neurofibrillary tangles (Yokoyama et al., 2022). Neuronal loss and tangles as well as synaptic loss, neuroinflammation and cognitive impairment are seen in PS19 mice overexpressing the microtubule-associated protein tau (*MAPT*) harboring the *MAPT-P301S* mutation linked to frontotemporal dementia, an AD-related disease, and these mice have been used for modeling AD-like tau pathology while plaque pathology is expectedly not seen (Yoshiyama et al., 2007; Takeuchi et al., 2011). Other models like the 3xTg mice carrying the AD-associated *APP-Swedish*, *MAPT-P301L* and *PSEN1-M146V* mutations demonstrate formation of Aβ plaques and neurofibrillary tangles along with neuroinflammation, synaptic dysfunction, cognitive impairment (Oddo et al., 2003; Caruso et al., 2013). Since these models overexpress fAD genes to non-physiological levels, several knock-in (KI) models have been established, such as the *App^NL-G-F^* or the *App^NL-F^* mice that harbor the Swedish and Beyreuther/Iberian *App* mutations with or without the Arctic *App* mutation (Saito et al., 2014; Sasaguri et al., 2022). Both *App^NL-G-F^* and *App^NL-F^* mice demonstrate more physiological levels of gene expression with Aβ pathology, neuroinflammation and cognitive impairment, but tangle pathology and neuronal loss are not seen (Saito et al., 2014).

While these and other mouse models have helped us to understand mechanistic links in AD, there are species-specific differences between mice and humans that have to be considered. For example, species-specific differences in the splicing of tau may limit the study of endogenous tau pathology in mice. The *MAPT* gene is spliced to generate 3R or 4R tau isoforms that include (4R) or lack (3R) exon 10, which encodes one of the four microtubule binding domains (Goedert et al., 1988, 1989a,b). Both 3R and 4R tau isoforms can include or lack exons 2 and 3, resulting in a total of six tau isoforms (Trabzuni et al., 2012). In the healthy adult human brain and in the brains of AD patients, the 3R/4R ratio is balanced, but in the adult mouse brain, only 4R tau is seen (Goedert et al., 1989a; Takuma et al., 2003). Such species-specific differences, the observation that only part of the AD neuropathology is modeled in many AD mouse models, and the fact that most AD mouse models represent models of fAD may be some of the reasons why clinical trials with promising results in mice have failed in patients with late-onset AD (Cummings et al., 2018; King, 2018; Ceyzeriat et al., 2020). They also indicate that a human cellular background may be needed to study human-specific disease mechanisms of AD and to develop novel therapeutic approaches.

In this context, human induced pluripotent stem cells (iPSCs) carry a strong potential for translational research. iPSCs resemble embryonic stem cells (ESCs) in their biological characteristics (Takahashi and Yamanaka, 2006; Takahashi et al., 2007) and have been widely used to model neurological diseases including AD and related dementias such as frontotemporal dementia (FTD; Lines et al., 2020; Kuhn et al., 2021; Qu et al., 2023). iPSCs originate from somatic cells, most commonly fibroblasts or peripheral blood mononuclear cells, modified to overexpress pluripotency-associated transcription factors such as *OCT4*, *KLF4*, *SOX2* and *c-MYC* (Takahashi and Yamanaka, 2006; Takahashi et al., 2007). Various published protocols allow efficient differentiation of human iPSCs or ESCs into cortical (Espuny-Camacho et al., 2013; Zhang et al., 2013), dopaminergic (Soldner et al., 2009; Cooper et al., 2010; Hargus et al., 2010; Kriks et al., 2011; Cooper et al., 2012; Sundberg et al., 2013; Kim et al., 2021) or motor neurons (Du et al., 2015; Castillo Bautista and Sterneckert, 2022). Other protocols have been developed to successfully generate microglia (Muffat et al., 2016; McQuade et al., 2018), astrocytes (Serio et al., 2013; Hallmann et al., 2017) or oligodendrocytes (Stacpoole et al., 2013; Douvaras et al., 2014; Ehrlich et al., 2017) *in vitro*. Thus, high numbers of brain cells can readily be generated from human donors - healthy individuals or patients – via iPSC intermediates, providing ample opportunities for drug or toxicity screens, cell replacement therapy or disease modeling. Differentiating iPSCs recapitulate developmental programs and differentially express genes according to their developmental stage. For instance, the fetal isoform of tau is produced earliest during neuronal differentiation of iPSCs as seen in the fetus, while all six tau isoforms, including the 4R tau isoforms, are expressed in mature iPSC neurons after several months of maturation *in vitro* (Iovino et al., 2015). Differentiating stem cell-derived neurons also upregulate products of APP processing (Koch et al., 2012; Bergström et al., 2016). iPSC-derived astrocytes express glutamate receptors and have the ability to take up glutamate and to propagate calcium waves (Hallmann et al., 2017), while iPSC-derived oligodendroglial cells express myelin components and can surround and myelinate axons (Ehrlich et al., 2017). iPSC-derived microglial cells are responsive to inflammatory cues and release pro-inflammatory cytokines upon stimulation (Abud et al., 2017). These functional maturation programs in iPSC-derived neuronal and glial cells are an important prerequisite for their application for drug screening, cell replacement or disease modeling purposes.

**Figure 1:**
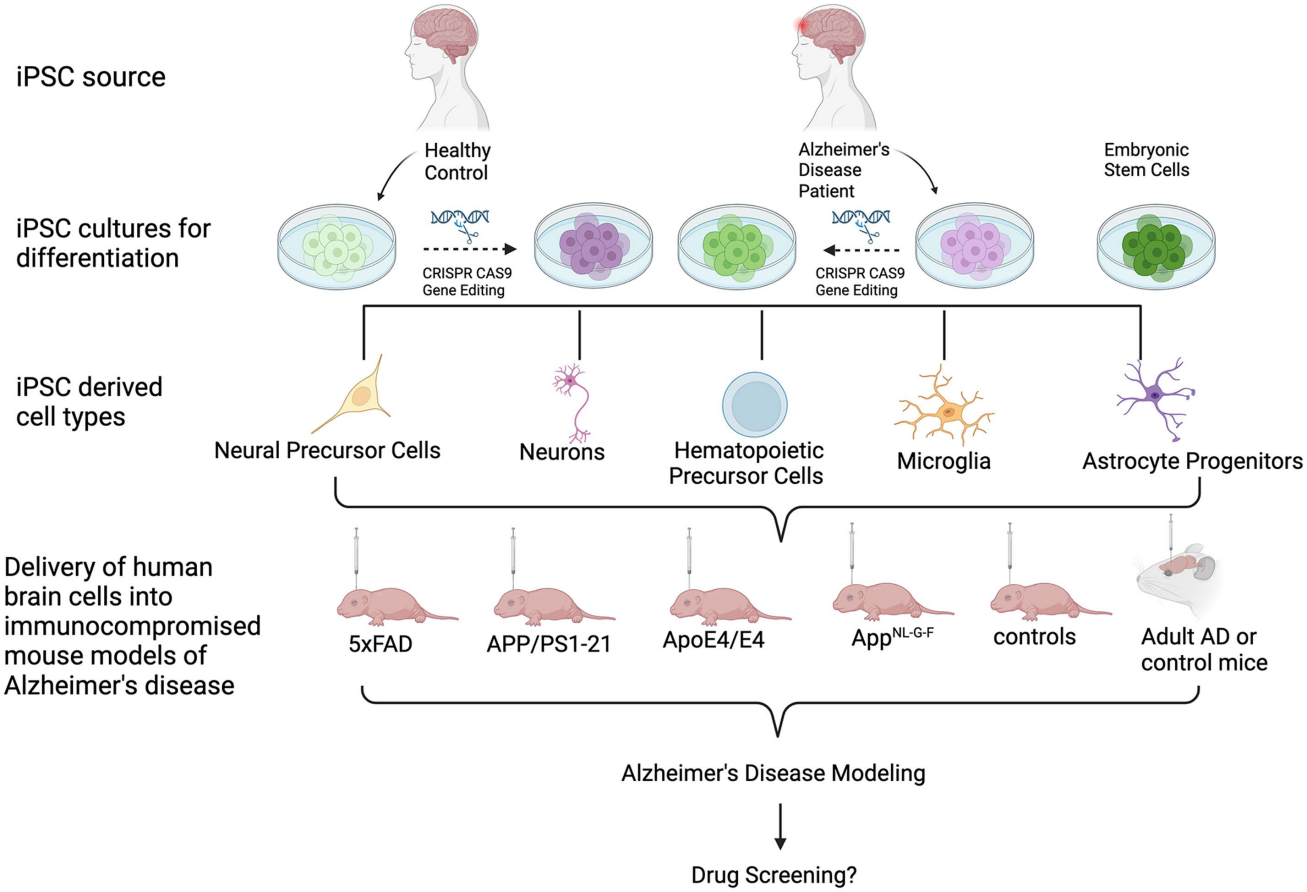
Schematic of the transplantation of human iPSC- and ESC-derived cells into mouse models of AD. From top to bottom: iPSCs are derived from healthy donors or AD patients. ESCs may also be used. Cultures can undergo gene editing to introduce or repair an AD-associated genetic mutation or risk gene. Cells are then differentiated into cell types of interest, followed by transplantation into immunocomprosmised mouse models of AD. Beyond modeling AD pathologies, this *in vivo* tool can be used for biomedical applications, such as investigating the efficacy of potential drugs or therapeutics.

Several groups have transplanted human ESC- or iPSC-derived neural cells into the brains of mice (Kriks et al., 2011; Reinhardt et al., 2013; Hargus et al., 2014), rats (Cooper et al., 2010; Hargus et al., 2010; Kriks et al., 2011; Sundberg et al., 2013; Wakeman et al., 2017; Song et al., 2020; Kim et al., 2021; Park et al., 2024) or primates (Kriks et al., 2011; Hallett et al., 2015; Wakeman et al., 2017), demonstrating successful engraftment and survival of human cells in these brains up to several months or, in the case of primates, even years after cell injection. Many of these reports have used human ESC- or iPSC-derived neural progenitor cells (NPC) or differentiated neurons for regenerative purposes, such a dopaminergic neurons for cell replacement in animal models of Parkinson’s disease (Hargus et al., 2010; Kriks et al., 2011; Wakeman et al., 2017). Human iPSC-derived neural cells have also successfully been injected into animal models of spinal cord injury (Shibata et al., 2023). Other reports applied cell transplantation to study neuronal cell type-specific connectivity (Espuny-Camacho et al., 2013) or the effect of the cellular origin prior to iPSC generation on cell survival and maturation *in vivo* (Hargus et al., 2014). Over the past few years, several groups have also injected healthy human or patient-derived iPSC-derived neuronal and glial cells into the brains of either healthy or diseased mice to model human-specific disease phenotypes in a more physiologic microenvironment. In this review, we provide an overview of studies that applied injections of differentiated human iPSCs into mouse brains to model AD (Figure 1; Table 1).

**Table 1:** Summary of human transplantation models of AD.

Cell type injected	Genotype of transplanted human cells	Host model	Age of mice during injection	Site of injection; cell number	Time point of analysis after injection	Phenotypes^*^	References
NPCs	WT (H9 hESC-derived)	APP/PS1-21 – NOD-SCID mice NOD-SCID mice as controls	Neonatal P0	Frontal cortex; 100,000 cells.	2, 4, 6 and 8 months	After injection into APP/PS1-21 mutation mice when compared with NOD-SCID controls: Increased numbers of reactive astrocytes and microglia (4 MPI). Pronounced dystrophic neurite pathology (4 MPI). Significant loss of human neurons with hyperphoshorylation of tau; abnormal tau conformational changes (6 MPI). Downregulation of genes related to neuron function (4–5 MPI, 8 MPI).	Espuny-Camacho et al. (2017)
	MAPT-10 + 16 (hiPSC-derived)	APP/PS1-21 – NOD-SCID mice; NOD-SCID mice as controls	Neonatal P0	Frontal cortex; 100,000 cells.	2, 4, 6 and 8 months	Similar phenotypes as described for human WT cells in AD mice with acceleration of 4R tau pathology (4 MPI).	
NPCs	WT (H9 hESC-derived)	*Rag2^−/−^/App^NL-G-F^* mice *Rag2^−/−^* mice as controls	Neonatal P1-P2	Frontal cortex; 100,000 cells.	2, 6 and 18 months	After injection into *Rag2^−/−^/App^NL-G-F^* mice when compared with *Rag2^−/−^* controls: Neuronal loss and neurodegenerative changes in human neurons (6 MPI). Formation of tangles and secretion p-tau181 and p-tau231 into blood (18 MPI). Necroptosis in transplanted neurons with upregulation of *MEG3* (6 MPI). Improved survival of human neurons through inhibition of necroptosis (6 MPI).	Balusu et al. (2023)
Neurons	APOE4/4 (hiPSC-derived) APOE3/3 (hiPSC-derived, isogenic to APOE4/4 hiPSC)	ApoE4/4-KI (E4KI) mice ApoE3/3-KI (E3KI) mice	7 months	Two hippocampal injections in each hemisphere; 20,000 cells per injection site; 80,000 cells.	7 months	Histology: Formation of plaque-like aggregates originating from both APOE4/4 and APOE3/3 neurons; APOE4/4 neurons produce more plaque aggregates in E4KI mice than in E3KI mice. Impaired phagocytosis of Aβ aggregates by E4KI microglia independent of APOE status of injected human neurons. snRNA-seq of isolated human cells: A. Human excitatory neurons: APOE4/4 neurons in E4KI mice exhibit dysregulation of genes related to synaptic function and calcium signaling. APOE3/3 neurons in E4KI mice exhibit dysregulation of pathways related to ribosome, spliceosome, and protein processing. B. Human inhibitory neurons: APOE3/3 neurons in E4KI mice exhibit dysregulation of 27 pathways related to mitophagy, autophagy, and apoptosis; increased expression of heat shock related proteins. APOE4/4 neurons in E4KI mice show dysregulation of pathways related to autophagy, longevity, protein degradation and secretion.	Najm et al. (2020)
HPCs	WT (hiPSC-derived)	5X-MITRG mice MITRG mice as controls	Neonate P0-P1	Cortical and ventricular; 62.5 k cells/injection 8 injections; 500,000 cells.	9 months; 10.5–12 months	After injection into 5X-MITRG mice, when compared with MITRG controls: Activated morphology of human microglia with upregulation of CD9, MERTK, TREM2 and APOE and occasional phagocytosis of fibrillar Aβ (9 MPI). Single cell RNA seq reveals shift in microglial sub-clusters and in human-specific DAM signature (10.5–12 MPI).	Hasselmann et al. (2019)
	WT (hiPSC-derived) *TREM2 R47H* (hiPSC-derived; isogenic to WT)	5X-MITRG mice	Neonate P0-P1	Cortical and ventricular; 62.5 k cells/injection 8 injections; 500,000 cells.	9 months	Comparing *TREM2 R47H* with WT cells: Reduced number of *TREM2 R47H* microglia around Aβ plaques.	
HPCs	WT + *TREM2-*KO as mixed cell suspension (hiPSC-derived)	MITRG mice	Neonate P2	Cortical and ventricular; 62.5 k cells/injection 8 injections; 500,000 cells.	6 months	Reduced migratory activity of *TREM2-*KO cells. Hypo-reactivity of *TREM2-*KO cells, remain in more homeostatic like state.	McQuade et al. (2020)
	WT + *TREM2-*KO as mixed cell suspension (hiPSC-derived)	5xfAD-MITRG mice	Neonate P2	Cortical and ventricular; 62.5 k cells/injection 8 injections; 500,000 cells.	6 months	*TREM2*-KO cells show impaired responsiveness to amyloid plaques. Hypo-reactivity of *TREM2*-KO cells, remain in more homeostatic like state. *TREM2*-KO cells show decreased ability to enact DAM associated transcriptional programs.	
HPC	WT (hiPSC-derived) *TREM2-R47H* (hiPSC-derived; isogenic to WT cells)	5x-hCSF1 mice	Neonatal P1-P2	Cortical and ventricular; 50 K cells/ injection 8 injections; 400,000 cells.	7 months	Reduced reactivity of *TREM2-R47H* microglia to Aβ plaques with reduced expression of CD9 and reduced secretion of APOE. Decreased lipid droplet accumulation in *TREM2-R47H* microglia. WT and *TREM2-R47H* microglia resemble atherosclerotic foam cells on a transcriptional level.	Claes et al. (2021)
iMGLs	WT (hiPSC-derived)	Rag2-5xfAD mice MITRG mice	N/A	Hippocampus; 100,000 cells.	2 months	Successful engraftment and migration along white matter tracts. Migration towards and surrounding Aβ plaques. Phagocytosis of fibrillar Aβ.	Abud et al. (2017)
iMGLs	WT (H9-hESC-derived)	*Rag2^−/-^Il2ry^−/-^hCSF1^KI^* mice	Neonatal P4	2 Locations in the forebrain; 200,000 cells.	8–10 weeks	Shift in transcriptional profile after application of oligomeric Aβ; transcriptional alteration different between human and mouse microglia.	Mancuso et al. (2019)
Astrocyte progenitor cells	APOE4/4 cells (hiPSC-derived) APOE3/3 (hiPSC-derived, isogenic to APOE4/4 hiPSC)	APP/PS1-21 – NOD-SCID mice NOD-SCID mice	Neonatal P0-P4	2 Locations in the forebrain; 200,000 cells.	5 months	Hypertrophy of processes surrounding plaques; cells also show atrophic features; changes independent of APOE status of injected cells.	Preman et al. (2021)

^*^Timepoints post injection where phenotypes first seen in mice are indicated within parenthesis. P(#); post-partum, followed by the number of days after birth. Apoe, Apolipoprotein E; APP/PS1-21, transgenic mouse line Tg (Thy1-APPSw, Thy1-PSEN1∗L166P) 21Jckr; DAM, disease associated microglia; FAD, familial Alzheimer’s disease; hCSF1, human colony stimulating factor 1; hESC, human embryonic stem cell; HPC, hematopoietic progenitor cell; iMGL, induced pluripotent stem cell-derived microglia; KEGG, Kyoto Encyclopedia of Genes and Genomes; KI, Knock-In; KO, Knock Out; MITRG, Rag2−/; -Il2ry−/−; M-CSFh; IL-3/GM-CSFh; TPOh; MPI, months post injection; NOD-SCID, immunodeficient NOD.CB17-Prkdcscid/J; N/A, details not available; NPC, neural precursor cell; RAG2, recombination activating gene 2; TREM2, triggering receptor expressed in myeloid cells 2; WT, wild type.

## Human ESC- and iPSC-derived neuron transplantation models of AD

Since neuronal tau and Aβ pathology as well as neuronal cell death are major substrates of AD pathology, many groups have studied disease phenotypes in cultured neurons from patients with fAD in comparison to healthy donor or gene-corrected control cells. AD iPSC-derived neurons carrying mutations in *APP*, *PSEN1* or *PSEN2* show an increased production of Aβ or an increased Aβ_42_/ Aβ_40_ ratio, as well as elevated levels of total tau and phosphorylated tau (p-tau), which accumulates in neurofibrillary tangles (Muratore et al., 2014; Ortiz-Virumbrales et al., 2017; Yang et al., 2017; Qu et al., 2023). Some of the additional disease phenotypes in AD iPSC-derived neurons included elevated oxidative stress, lysosomal dysregulation and increased excitability (Martin-Maestro et al., 2017; Li et al., 2018; Ghatak et al., 2019; Qu et al., 2023). Interestingly, formation of neurofibrillary tangles or Aβ plaques is typically not seen in conventional two-dimensional cultures of AD-derived iPSC neurons, but filaments of aggregated tau can appear when AD neurons are cultured in three-dimensional systems, as demonstrated with AD neurons overexpressing mutant *APP* or *PSEN1* (Choi et al., 2014). Three-dimensional organoids and three-dimensional hippocampal spheroids composed of AD iPSC-derived neural cells also showed increased levels of p-tau, Aβ pathology and synaptic dysfunction, the latter of which is an early feature of AD pathology (Raja et al., 2016; Gonzalez et al., 2018; Lin et al., 2018; Pomeshchik et al., 2020; Zhao et al., 2020). These findings highlight that a three-dimensional microenvironment provides beneficial cues to elicit AD pathological changes in AD iPSC-derived neurons.

The transplantation of human neurons into the brains of mice provides additional advantages over cultured neurons since the brain microenvironment provides important features lacking in cell culture systems. In particular, it allows to study graft-host interactions in a heterogeneous cellular context, the role of neuroinflammation or the function of the vasculature on human neurons and glial cells over several months in the adult brain, potentially giving novel insights in cell autonomous versus non-cell autonomous mechanisms of disease development.

Such non-cell autonomous effects could be mediated by Aβ, since deposition of Aβ precedes tau propagation, neuronal depletion, and clinical manifestations of AD by several decades and may thus be an early driver of AD progression (Dubois et al., 2014; Tolar et al., 2021). Espuny-Camacho et al. studied the effects of environmental Aβ plaques on healthy human neurons by transplanting human ESC-derived cortical neural precursors into the brains of neonate mice (Espuny-Camacho et al., 2017; Table 1). In one cohort, immunodeficient NOD-SCID mice were used as host controls, while another group of mice represented APP/PS1-21 mice (Radde et al., 2006) crossed with NOD-SCID mice (Shultz et al., 1995) to generate immunodeficient AD mice with Aβ pathology. In AD mice, human neurons were not only exposed to Aβ plaques but also to significantly increased numbers of astrocytes and microglia with reactive phenotypes. At 4 months after cell injection, increased numbers of dystrophic neurites were found around plaques within the grafts with abnormal accumulation of presynaptic and axonal proteins and reduction of human dendritic and postsynaptic proteins, as similarly seen in AD patient brains (Brion et al., 1991). These changes reflected signs of neurodegeneration that were not observed in the control mice. At 6 months post transplantation, there was a significant loss of human neurons with ultrastructural signs of necrosis in AD mouse brains but not in control mice. This difference in human cell numbers was not observed at 2 months post transplantation, before plaques develop in AD mice, and suggested that the Aβ deposits are toxic to human neurons and represent the driving factor of degeneration of this cell type. At 8 months after cell injection, human neurons in AD mice exhibit tau pathology with positivity for AT8 (p-tau) and for MC1, indicating tau forms with pathological conformation (Jicha et al., 1997; Espuny-Camacho et al., 2017). However, while abnormal accumulation of straight filaments was seen, no definite tangle pathology was noted in human neurons in AD mice. In line with these histological changes, bulk RNA sequencing on 8 month-old human grafts revealed significant upregulation of genes related to cell death and a significant downregulation of genes involved synaptic transmission when compared to human grafts in control mouse brains. Similar phenotypes of neuronal death and neurodegeneration were also observed when FTD iPSC-derived neural cells carrying the intronic *MAPT-10 + 16* mutation were used for cell injection instead of healthy control cells. However, an accelerated expression of 4R tau isoforms was noted in these grafts, consistent with the function of this *MAPT* mutation in promoting 4R tau isoforms (Espuny-Camacho et al., 2017). Notably, this study also showed that neuronal loss and neurodegeneration were not observed in the surrounding mouse tissue or in grafts composed of mouse ESC-derived neurons. In addition, MC1 pathological conformational changes in tau were not seen in the AD mouse host tissue (Espuny-Camacho et al., 2017). These findings highlighted that the effects on neurodegeneration were species-specific and that it was important to use human cells for cell injection to induce a neurodegeneration phenotype *in vivo*.

The same group later showed that human ESC-derived neurons, but not mouse neurons, develop tangle pathology 18 months after injection into immunocompromised *Rag2^−/−^*/App^NL-G-F^ (App^tm3.1Tcs^/App^tm3.1Tcs^) knock-in mice, which also have pronounced Aβ pathology (Balusu et al., 2023; Table 1). This pathology was paralleled by secretion of the soluble biomarkers p-tau181 and p-tau231 into the bloodstream. Neuronal loss with presence of plaque-associated tau as well as AT8- and MC1-positivity was seen, that already appeared 6 months after cell injection in AD mice but not in immunocompromised *Rag2^−/−^* control mice. This study also showed that cell death in the human grafts is related to increased necroptosis induced by the long noncoding RNA MEG3, identified through RNA sequencing of grafted cells 6 and 18 months after injection. MEG3-induced cell death could be rescued after treatment with necroptosis inhibitors, and inhibition of necroptosis prevented human neuronal cell death after transplantation into AD mice (Balusu et al., 2023). MEG3 is also upregulated in neurons of AD patients, highlighting the strong value of using human-mouse chimera models to identify potential therapeutic targets in AD and to address human-specific vulnerability in AD.

Human iPSCs have also been applied to study the impact of the apolipoprotein (Apo) E4 genotype on AD pathological changes in human neurons *in vivo*. ApoE4 is the strongest genetic risk factor for AD and supports the production of Aβ and p-tau in human neurons (Wang et al., 2018). In a study by Najm et al. either APOE4/4 (E4/4) or isogenic APOE3/3 (E3/3) human iPSC-derived neurons were injected bilaterally into the hippocampus of either ApoE3/3-KI (E3KI) or ApoE4/4-KI (E4KI) mice to study cell-autonomous and non-cell-autonomous mechanisms and in particular the effects of either human transplant-derived APOE4 (endogenous) or environmental mouse-derived apoE4 (exogenous) on neuronal pathology in human neurons 7 months after cell injection (Najm et al., 2020; Table 1). Both E3/3 and E4/4 human neuronal grafts were composed of excitatory and inhibitory neurons, which showed subtype-specific changes in differential gene expression profiles depending on whether they were injected into E3KI or E4KI mice. In fact, snRNA-seq on human grafts revealed that human excitatory neurons showed the most pronounced dysregulation of genes, including genes linked to synaptic function and regulation of calcium signaling, when the E4/4 genotype was present in both the transplanted neurons and in the host. In contrast, human E3/3 excitatory neurons showed only few, compensatory gene expression changes in E4KI mouse brains. Human inhibitory neurons in transplants were more susceptible to ApoE4 and, unlike excitatory neurons, responded to both endogenous human neuron-derived and exogenous mouse-derived Apoe4 with an enrichment of genes linked to UPR, oxidative stress and RNA degradation, consistent with a previously reported increased susceptibility of inhibitory neurons to ApoE4 (Wang et al., 2018; Najm et al., 2019). Interestingly, the injection of either E3/3 or E4/4 human neurons resulted in the formation of human neuron-derived Aβ plaque-like aggregates in both E3KI or E4KI mice, which typically lack plaque formation (Huang, 2010). Plaque formation was further stimulated in the presence of mouse-derived exogenous ApoE4, and there was also impaired phagocytosis of Aβ aggregates by microglia in E4KI mice independent of the ApoE status of injected human neurons (Najm et al., 2020). Overall, these human-mouse chimeric transplantation models demonstrate that human excitatory and inhibitory neurons show a differential response to Apoe4 *in vivo* and that both human neurons as well as endogenous and environmental Apoe4 significantly contribute to pathological changes in this animal model of AD.

## Human iPSC-derived microglia transplantation models of AD

Microglia have emerged as an important cell type in several neurodegenerative diseases including AD. As the resident immune cell of the brain, microglia are able to detect and respond to stimuli within their microenvironment through cytokine release, to mobilize an immune response to protect the brain while recruiting peripheral immune cells like T cells, and to help to clear their surroundings by phagocytosing debris and dead cells (Hickman et al., 2013; Butler et al., 2021; Chen et al., 2023). Microglia also play an important role in neurogenesis, neural plasticity and in synaptic pruning (Paolicelli et al., 2011; Shigemoto-Mogami et al., 2014). In AD, microglia closely associate with neuritic plaques (McGeer and McGeer, 1999), secrete proinflammatory factors (Heneka and O'Banion, 2007) and phagocytose Aβ (Frautschy et al., 1998; Bolmont et al., 2008). The activation of microglia can occur through Aβ sensing and involves P2X_7_ receptor (Sanz et al., 2009) as well as Toll-like receptors TLR2 and TLR4 (Lotz et al., 2005). Microglia can also become activated via the cyclic GMP-AMP synthase (cGAS)-Stimulator of interferon genes (STING) pathway in response to tau binding to polyglutamine protein binding 1 (PQBP1) in microglia (Jin et al., 2021). Additionally, tau filament-containing neurons aberrantly expose phosphatidylserine residues that induce microglia to phagocytose them (Brelstaff et al., 2018). Besides their ability to phagocytose tau, reactive microglia promote tau pathology and contribute to the spread of pathological tau in the brain (Gorlovoy et al., 2009; Asai et al., 2015; Maphis et al., 2015; Hopp et al., 2018; Pascoal et al., 2021; Wang et al., 2022).

Genetic studies have identified risk genes such as *APOE*, *TREM2* (Triggering Receptor Expressed in Myeloid Cells 2), and *CD33*, all genes with expression patterns that are highly enriched within the microglial population (Bertram et al., 2008; Hollingworth et al., 2011; Naj et al., 2011; Guerreiro et al., 2013; Jonsson et al., 2013; Griciuc et al., 2019). For instance, TREM2 is almost exclusively expressed in the brain by microglia and has been widely studied in animal and cell culture models of AD. TREM2 may have a protective role in AD since it aids in the migration and aggregation of activated microglia around Aβ plaque deposits and in their uptake through phagocytosis (Xiang et al., 2016). Loss of function mutations in *TREM2*, like the R47H mutation, leads to a several fold increased risk for AD (Sayed et al., 2021).

Animal models of AD have provided valuable insights into microglial cell biology, but mouse microglia, similar to mouse neurons, are unable to fully recapitulate human cell biology due to species-specific differences such as differences in gene expression profiles and inflammatory response (Kodamullil et al., 2017). Several AD-GWAS risk genes including CD33 do not have functionally similar murine orthologs, limiting the study of certain AD risk genes in mouse microglia (Mancuso et al., 2019). The use of primary microglia isolated from human brain samples could represent an alternative, but short culture longevity and changes in gene expression and biological function upon isolation exhibit additional limitations to this approach (Gosselin et al., 2017).

Several protocols have been developed to derive human microglia-like cells from human iPSCs. They can be differentiated through an intermediate stage of hematopoietic stem cells (HPCs; McQuade et al., 2018), while embryoid body (EB)-based (Muffat et al., 2016; Douvaras et al., 2017), EB-neuron-co-culture based (Haenseler et al., 2017) or astrocyte-coculture based (Pandya et al., 2017) microglia differentiation protocols have also been established. These cell culture studies showed that human iPSC-derived microglia resemble endogenous human microglia and recapitulate the functional activities of the latter. They can secrete pro-inflammatory cytokines such as TNF-α, IL-1α and IL-6 upon inflammatory stimulation with LPS, IL1-β or interferon-γ (Abud et al., 2017). They have the ability to migrate and to phagocytose human synaptosomes in culture, which suggests they have the ability to participate in synaptic pruning (Abud et al., 2017). In addition, human iPSC-derived microglia are able to phagocytose fibrillar Aβ as well as brain-derived tau oligomers resulting in an upregulation of a subset of tested AD-GWAS risk genes including *ABCA7*, *CD33* and *TREM2* or *CD2AP*, respectively (Abud et al., 2017).

Improved maturation of human iPSC-derived microglial cells was also achieved through co-coculture with human iPSC-derived organoids, which were penetrated by the microglial cells (Park et al., 2023). In these organoids, the human microglial cells showed an activated morphology, expressed increased levels of *P2RY12*, *CX3CR1* and *SALL1* but decreased levels of *TMEM119* compared to human iPSC-derived microglial cells in two-dimensional cultures, and they promoted functional maturation of human NPCs into neurons with increased axonogenesis and overall reduction of organoid size. Interestingly, improved neurogenesis from NPCs was mediated through uptake of cholesterol and its esters released by the co-cultured microglia through PLIN2-positive lipid droplets which were also identified within microglia in the developing mouse and human brain (Park et al., 2023). These findings highlight that human iPSC-derived microglia interact with neural cells in a three-dimensional context and that a three-dimensional microenvironment favored functional maturation of human iPSC-derived microglial cells.

Human iPSC-derived microglia have been transplanted into the mouse brain with the aim to recapitulate the complexities and heterogeneity of microglial behavior in an *in vivo* system and to study the function of human microglia in an AD microenvironmental context (Table 1). To this end, several methods have been developed to further improve the engraftment of microglia, such as the ablation of endogenous microglia prior to cell injection with compounds that inhibit CSF1R (Mancuso et al., 2019; Xu et al., 2020; Fattorelli et al., 2021; Parajuli et al., 2021), and, most importantly, the application of immunocompromised mice with hM-CSF1-KI including MITRG mice (Rongvaux et al., 2014), that express humanized CSF-1 to support human microglia survival, since human CSF1R signaling cannot be fully activated by the murine CSF1 ligand (Rathinam et al., 2011; Elmore et al., 2014). One study reports trans-nasal delivery of human iPSC-derived microglia, which then penetrated the cribriform plate to migrate to the cortex, hippocampus and cerebellum keeping the blood–brain barrier intact (Parajuli et al., 2021). However, to better control the distribution and survival of human microglia, HSCs or microglia were mostly injected directly into the brains of mice. As shown by several groups, the injection of cells into the developing postnatal brain further improved their distribution with expansive and highly efficient engraftment (Hasselmann et al., 2019; Mancuso et al., 2019; Svoboda et al., 2019; Xu et al., 2020).

Using these methods, human iPSC-derived HPCs or human iPSC-derived microglia were successfully transplanted into the cortex, hippocampus, white matter tracts or ventricle of neonatal or adult mice expressing humanized CSF1 (Abud et al., 2017; McQuade et al., 2018; Hasselmann et al., 2019; Mancuso et al., 2019; Svoboda et al., 2019; Xu et al., 2020; Fattorelli et al., 2021), leading to successful survival, migration, engraftment and maturation of human microglia with a ramified morphology, resembling endogenous quiescent microglia. Transplanted human iPSC-derived microglia perform important cell functions. They extend and retract their processes to survey their microenvironment, and they react towards brain injury with the phagocytosis of degenerating neurons (Hasselmann et al., 2019). RNA sequencing analyses of isolated human microglia revealed that they resemble *in vivo* human microglia and that they recapitulate their heterogeneity (Hasselmann et al., 2019; Mancuso et al., 2019; Svoboda et al., 2019; Xu et al., 2020). Transplanted human iPSC-derived microglia are responsive to inflammatory cues such as LPS (Hasselmann et al., 2019) and they respond to an intraventricular injection of oligomeric Aβ with an altered transcriptome profile which differs from the gene expression profile in endogenous mouse microglia exposed to the same Aβ treatment (Mancuso et al., 2019).

Human iPSC-derived microglia were injected into the hippocampus of Rag-5xfAD mice that overexpress fAD-associated mutant *APP* (Swedish, Florida, London) and mutant *PS1* (M146L and L286V) resulting in robust Aβ pathology (Oakley et al., 2006; Marsh et al., 2016; Abud et al., 2017; Table 1). Two months after cell injection, human iPSC-derived microglia survived and engrafted in AD brains and migrated along white matter tracts. In addition, many human microglia surrounded Aβ plaques, sent their processes towards these aggregates and started to phagocytose fibrillar Aβ (Abud et al., 2017). By 9 months after injection into 5X-MITRG AD mice, plaque-associated human microglia express numerous markers of disease-associated microglia (DAM) including CD9, TREM2 and APOE (Hasselmann et al., 2019). Single cell RNA-sequencing on human microglia in AD mouse brains versus MITRG control brains revealed a shift in the microglial subpopulations with larger DAM and MHCII clusters, presence of a secretory cluster and reduction of an interferon cluster on AD brains. Notably, the authors also detected a human-specific microglial response to Aβ when they compared differentially expressed genes in the DAM versus homeostatic clusters in transplanted human microglia with the DAM signature in mouse microglia (Keren-Shaul et al., 2017; Hasselmann et al., 2019). This approach revealed that only a minority of DAM genes overlapped between both species, while it identified new, human-specific Aβ responsive genes, highlighting the strong value of such mouse-human chimeric mouse model to study human-specific microglial function in AD (Hasselmann et al., 2019).

Transplantation models were also established to study the role of TREM2 in human microglia *in vivo* (Table 1). Human iPSCs were genetically engineered to generate isogenic cells carrying the AD-associated *TREM2 R47H* mutation, followed by *in vitro* differentiation and transplantation of HPCs into the 5x-hCSF1 or 5x-MITRG mouse models of AD and analysis 7 or 9 months after cell injection, respectively (Hasselmann et al., 2019; Claes et al., 2021). TREM2 is a lipid sensing receptor, and the authors found that both transplanted wildtype and *R47H* microglia around Aβ plaques contain lipid droplets and resemble atherosclerotic foam cells at a transcriptional level (Claes et al., 2021). However, the number of human *R47H* microglia around Aβ plaques was significantly reduced, as similarly described in the brains of human *R47H* carriers (Yuan et al., 2016; Hasselmann et al., 2019). Impaired migration of *R47H* microglia towards Aβ plaques was paralleled by reduced reactivity with reduced expression of the DAM marker CD9, reduced secretion of APOE and reduced accumulation of lipid droplets (Claes et al., 2021). These findings indicated impaired *R47H*-associated microglial function *in vivo* with a failure to properly initiate programs of activation.

In a related study, McQuade et al. compared human *TREM2*-deficient (*TREM2*-KO) and WT iPSC-derived microglia *in vitro* and *in vivo* (McQuade et al., 2020; Table 1). Here, cultured *TREM2*-KO microglia were hypersensitive to starvation-induced cell stress and showed impaired phagocytosis of APOE, fibrillar Aβ and synaptosomes. When injected as mixed suspension of WT / *TREM2*-KO HPCs into the brains of neonatal MITRG control and 5xfAD-MITRG AD mice, WT microglia displayed robust response to Aβ plaque pathology with an aggregation around plaque complexes at 6 months after cell injection. Conversely, while the *TREM2*-KO microglia appeared unresponsive to plaque pathology and displayed impaired migration. In addition, single cell RNA sequencing of transplanted cells revealed a shift of microglial subclusters towards homeostatic, less reactive profiles in *TREM2*-KO microglia, which also failed to acquire a DAM signature when injected into AD mice, further supporting the notion that *TREM2* knock out traps microglia into a homeostatic state (McQuade et al., 2020).

## Human iPSC-derived astrocyte transplantation models of AD

In the healthy brain, astrocytes provide energy substrates and neurotrophic factors to neurons, maintain homeostasis of extracellular ions and transmitters, influence synaptic transmissions, and modulate the permeability of the blood–brain barrier (Abbott et al., 2006; Mahmoud et al., 2019; Siracusa et al., 2019). Upon injury to the CNS, astrocytes have the ability to enact robust responses resulting in astrogliosis characterized by astrocyte recruitment, proliferation and activation (Sofroniew, 2014).

In AD, reactive astrocytes are found near Aβ plaques (Verkhratsky et al., 2019) and express proteases like MMP-2 and MMP-9 involved in the enzymatic clearing of Aβ (Kamat et al., 2014). An increased expression of these metalloproteinases is also seen in astrocytes that aggregate around Aβ plaques in AD mouse models (Verkhratsky et al., 2019). Transcriptome studies of astrocytes in AD mouse models demonstrate an upregulation of ‘defense response’ genes and a downregulation of genes related to synaptic transmission and neurogenesis (Monterey et al., 2021). APOE is one of the AD risk genes that is primarily expressed in astrocytes in the brain, and the expression of Apoe4 is associated with a decreased ability to clear Aβ plaques (Baitsch et al., 2011; Zhu et al., 2012). AD-associated astrocytes also secrete inflammatory cytokines like INF-γ, TNF-α, and IL-1β (Monterey et al., 2021), which may not only exacerbate neurotoxicity in degenerative diseases, but also stimulate nearby cells in the microenvironment, like microglia, to promote neuroinflammation. Moreover, accumulation of tau can also occur in hilar astrocytes of the hippocampus, potentially contributing to the propagation of tau (Richetin et al., 2020).

When studying these cells, it is important to note that there are substantial differences between human and rodent astrocytes. For instance, human astrocytes are larger than mouse astrocytes, they differ in the activation of signaling pathways, and they react differently to inflammatory stimuli (Han et al., 2013; Tarassishin et al., 2014; Degl'Innocenti and Dell'Anno, 2023). These species-specific differences in astroglial function highlight the need to use human cell-based models to accurately study the biology and pathology of this cell type in more detail.

Multiple groups have reported successful differentiation of astrocytes from human iPSCs including those from healthy donor and from patients with AD and related tauopathies (Hallmann et al., 2017; Oksanen et al., 2017; Tcw et al., 2017; Zhao et al., 2017; Lin et al., 2018; Perriot et al., 2021; Tcw et al., 2022). For instance, Zhao et al. differentiated astrocytes from APOE4/4 or APOE3/3 iPSCs to investigate their protective effects on co-cultured neurons. Viability assays showed that the co-culture of neurons with APOE4/4 astrocytes resulted in significantly lower neuronal viability than the co-culture with APOE3/3 (Zhao et al., 2017). Similarly, cultured patient iPSC-derived astrocytes carrying the FTD-associated *MAPT-N279K* mutation rendered previously healthy human neurons more susceptible to oxidative stress and induced gene expression changes in these neurons that were linked to apoptosis and cellular stress (Hallmann et al., 2017). These findings not only illustrate the capacity of astrocytes to shape neuronal survival, but they also highlight that human iPSC-derived astrocyte models can be used to analyze cell interactions and non-cell-autonomous disease mechanisms.

Cell transplantation studies with human glial progenitor cells have demonstrated successful engraftment of injected cells with migration throughout the mouse brain (Windrem et al., 2004, 2008; Han et al., 2013). For example, human fetal glial progenitor cells survive after injection into the neonatal mouse brain and continuously populate the brain over time, resulting in the presence of human astrocytes in the hippocampus, cortex, amygdala, thalamus, and neostriatum by 12–20 months of age (Han et al., 2013). These human astrocytes integrate into the mouse environment, they extend processes, and they exhibit functional electrophysiological properties as demonstrated on acute hippocampal slices. Furthermore, they participate in Ca^2+^ signaling and propagate calcium waves, which were about 3-fold faster than those in mouse astrocytes. Notably, the injection of human glial progenitor cells resulted in significantly enhanced long-term potentiation and improved learning, which was not seen in non-injected mice or in mice that had received mouse glial cells, again highlighting species-specific differences in biological function of glial cells *in vivo* (Han et al., 2013).

Human iPSC-derived astrocyte progenitor cells were also injected into the brains of neonatal mice resulting in the formation of different morphological astrocyte subtypes including interlaminar, protoplasmic, fibrillar and varicose-projection astrocytes, which are typically seen in the human brain (Preman et al., 2021; Table 1). Notably, many of these human astrocytes changed their appearance when injected into the brains of neonatal, immunocompromised APP/PS1-21 AD mice. Five months after cell injection, the human iPSC-derived astrocytes responded to the presence of Aβ plaques with the formation of hypertrophic profiles with thick processes as well as with the formation of atrophic profiles, both of which were also observed in the human AD brain (Preman et al., 2021). Interestingly, these Aβ-induced morphological changes in astrocytes were independent of an APOE4/4 or APOE3/3 allelic background in transplanted cells.

## Discussion

Recent advances in human iPSC technology with derivation of human neurons and glial cells have provided novel opportunities to ask important questions about human neurobiology and neurological diseases including AD. As useful as murine cells are in studying brain cell types, there are major species-specific biological differences that need to be considered, such as differences in gene expression programs, morphology, and inflammatory responses (Kodamullil et al., 2017; Hasselmann et al., 2019; Xu et al., 2020; Degl'Innocenti and Dell'Anno, 2023). The use of human cells circumvents these incongruencies and resulted in the discovery of various disease phenotypes and their regulation in patient-derived neurons and glial cells, as performed, for instance, to model AD and related dementias (Lines et al., 2020; Kuhn et al., 2021; Qu et al., 2023).

The scope of this developing field becomes even wider with cell transplantation to place human neuronal and glial cells into a brain microenvironment, which recapitulates important aspects of human disease. The brain harbors immense complexity and heterogeneity, and although *in vitro* culture platforms are instrumental for disease modeling, they are at this point still unable to encompass the diverse cellular interactions of the brain. As a few examples, the neuronal milieu promotes the maturation of microglia (Zhao et al., 2022), microglia modulate neurogenesis (Shigemoto-Mogami et al., 2014), and capillary-associated microglia regulate the structure and function of blood vessels within the brain (Bisht et al., 2021). Exposure of human cells to this highly organized microenvironment with its complex cell–cell interactions and various soluble factors provides important advantages for disease modeling, as it promotes increased cellular maturation and integration into functional circulatory systems, which in turn helps to better characterize phenotypic and functional differences between healthy control and patient cells.

Prior to transplantation, undifferentiated or differentiated human iPSCs can be genetically engineered to introduce or correct disease-associated gene mutations or disease-associated risk genes. At the same time, various animal models can be applied as host to test human-specific responses to pathological substrates such as Aβ plaques or to AD risk genes such as Apoe4 in animal models of AD. Important progress has also been made by generating humanized mice expressing the human sequences of Aβ or *MAPT* as *in vivo* models of tauopathy (Saito et al., 2014; Hashimoto et al., 2019; Saito et al., 2019; Baglietto-Vargas et al., 2021; Borcuk et al., 2022; Sasaguri et al., 2022). In contrast to most AD mouse models, humanized knock-in mice express human genes at physiological levels since human gene expression is driven by endogenous mouse promoters. Mice with humanized wildtype Aβ, aged to 18 to 22 months, show increased production of insoluble Aβ, impairment of cognition and synaptic plasticity as well as decreased hippocampal volume (Baglietto-Vargas et al., 2021). Humanized *MAPT* knock-in mice express all 6 tau isoforms including both 3R and 4R tau in contrast to the normal adult mouse brain, which only expresses 4R tau isoforms (Takuma et al., 2003), and they demonstrate a physiological distribution of tau within axons (Saito et al., 2019). When injected with tau protein extracted from AD patients, these *MAPT* knock-in mice demonstrate an accelerated propagation of the pathological human AD tau compared to wildtype mice expressing only murine tau, and tau propagation was further accelerated in the presence of mutant *APP* (Saito et al., 2019). These findings indicate that pathological human tau interacts better with human than with mouse tau and thus highlight species-specific differences in tau propagation. Additional humanized mouse strains with mutant *MAPT* have been generated (Sasaguri et al., 2022) and could be used as alternative hosts for cell injection studies. These gene modification and cell transplantation strategies in combination with histology, bulk and single cell RNA sequencing technologies provide important tools to analyze both cell-intrinsic and cell-extrinsic disease mechanisms in transplanted human cells. They also offer novel opportunities to model human-specific genetic aspects of neurodegenerative diseases including AD.

Despite these many exciting possibilities, there are also challenges and limitations when using human-mouse chimeric mouse models for disease modeling. Firstly, neurodegenerative diseases like AD predominantly affect an older patient population, mostly patients over the age of 65 (Alzheimer’s Association, 2012). On the other hand, the lifespan of the iPSC-derived cell transplants is limited by the lifespan of the host animal. Laboratory mice live an average of 26–30 months, in the case of immunocompromised mice usually shorter, and are unable to capture this aspect of human aging. Introducing cell stress or overexpressing ageing-accelerating genes in cells prior to transplantation could represent a way to address this limitation (Miller et al., 2013; Hartmann et al., 2023). Secondly, the distribution of transplanted human cells in the mouse brain may vary from cell line to cell line and appears to depend on the age of mice upon transplantation. For instance, when fully differentiated cells are transplanted into adult mouse brain, the cells may tend to aggregate in close proximity to the needle tract. This is not necessarily a disadvantage, as experiments that aim at studying human brain cells in smaller focal areas or those that involve the microdissection of localized human grafts for single cell analysis, might benefit from this approach. However, a wider distribution of human cells might be beneficial to better study graft-host interactions or the effects of human cells on behavioral outcomes in these mice. Several groups have successfully addressed this limitation and found that the transplantation of human HPCs, microglial cells or astrocyte progenitor cells into young neonatal mouse pups results in widespread engraftment of human cells throughout the brain (Hasselmann et al., 2019; Mancuso et al., 2019; Svoboda et al., 2019; McQuade et al., 2020; Xu et al., 2020; Claes et al., 2021; Preman et al., 2021). Thirdly, as outlined in this review, immunocompromised mice with or without AD pathology have been used for cell transplantation to protect the human cells from being rejected by mouse inflammatory cells. These mice lack an adaptive immune system, limiting the study of B, T and natural killer cells on human cell function in these chimeric animals. To address this limitation, xenograft studies have utilized immunocompetent mice treated with immunosuppressive cocktails around the time of transplantation to prevent graft rejection (Najm et al., 2020). The application of mouse strains with completely humanized immune system with preserved B and T cell populations may provide an additional alternative that would also allow to study the role of the adaptive immune system on human-specific disease mechanisms in chimeric mice. The ability to genetically manipulate human immune system components in mouse strains introduces an additional new advantage, as xenografts in mice with or without these modified components could be compared to study downstream effects on neurodegenerative phenotypes.

Since human iPSCs and their derivatives can readily be modified to express cell-type-specific or activity-dependent fluorescent reporter constructs, such reporter brain cells could be applied in chimeric mice to gain important information about the dynamics of neurodegenerative or disease-modifying programs within human cells. As an example, human microglia are equipped with a sensome, an assortment of receptors capable of inducing cell activation in response to environmental stimuli (Hickman et al., 2013). Human iPSC-derived microglia could be engineered to fluoresce upon activation of purinergic signaling, which has been linked to important AD-associated functions such as the release of pro-inflammatory cytokines and the degradation of Aβ plaques (Cieslak and Wojtczak, 2018). Following these cells in the brain through live cell imaging could provide novel insights into the temporal and spatial dynamics of microglial activation during disease development and progression. These dynamic functions could also be tested in transplanted human microglia that express variants or lack the expression of AD susceptibility genes, for example *CD33* and *BIN1 (Bridging INtegrator-1)*, as similarly shown for *TREM2* (Hasselmann et al., 2019; McQuade et al., 2020; Claes et al., 2021). CD33 is a member of the sialic acid-binding immunoglobulin-like lectins, and CD33 levels as well as numbers of CD33-expressing microglia are increased in the AD brain (Griciuc et al., 2013). Since *CD33* does not have a clear mouse ortholog (Mancuso et al., 2019), proposed transplantation studies using fluorescent human iPSC-derived microglia with altered *CD33*, either derived from patients or genetically engineered, could provide an opportunity to learn more about AD-associated functions of *CD33*, especially about its protective variant (Hollingworth et al., 2011; Naj et al., 2011; Griciuc et al., 2013). *BIN1,* the second most significant AD susceptibility gene in late-onset AD after *APOE* (Harold et al., 2009; Lambert et al., 2009; Seshadri et al., 2010) is expressed in neurons, microglia, and particularly in oligodendrocytes, and appears in different isoforms in the brain (Adams et al., 2016; De Rossi et al., 2016; Lambert et al., 2022). One of these *BIN1* isoforms, isoform 1, induces an accumulation of endosomal vesicles and neurodegeneration through early endosome defects, as recently demonstrated in a study applying the drosophila model and *BIN1*-deficient human iPSC-derived neurons with and without overexpression of *BIN1* isoform 1 (Lambert et al., 2022). Since enlarged early endosomes have been described in AD neurons, and since endosomal pathway activation represents an early response in AD (Cataldo et al., 2000), it would be very interesting to assess the dynamics and cell autonomy of *BIN1* isoform-mediated neurotoxicity in transplanted AD patient-derived or *BIN1*-engineered iPSC-derived neurons as well as *BIN1* isoform-driven effects in stem cell-derived human microglia and especially oligodendrocytes in AD chimeric brains using stress-, activation- or myelination-specific fluorescent reporter constructs.

The study of oligodendrocytes is relevant since oligodendrocytes are also altered in AD (Rodriguez et al., 2016; Nasrabady et al., 2018). Oligodendrocytes produce myelin, provide neurotrophic support to neurons, stabilize neuronal connectivity and can inhibit neurite outgrowth (Schwab, 1990). White matter abnormalities characterized by hyperintensities in imaging studies have been demonstrated in patients with AD and may be present even years before the onset of symptoms (Nasrabady et al., 2018). These abnormalities are associated with axonal loss and demyelination (McAleese et al., 2017). Reduced numbers of Olig2-positive cells in the postmortem human AD cortex have been reported (Behrendt et al., 2013), and myelin loss with reduced amounts of myelin basic protein (MBP), myelin proteolipid protein, cyclic nucleotide phosphohydrolase and cholesterol have been identified in postmortem AD brains (Roher et al., 2002; Benitez et al., 2014). While these changes may occur secondary to cortical pathology and neuroinflammation, it is also likely that increased oxidative stress, excitotoxity and calcium and iron dyshomeostasis in oligodendrocytes contribute to the pathogenesis in AD (Bartzokis, 2011; Rodriguez et al., 2016; Nasrabady et al., 2018). Oligodendrocytes can successfully be differentiated from human iPSCs and have the ability to myelinate axons *in vitro* and *in vivo*, as observed 16 weeks after injection of human oligodendroglial cells into the brain and spinal cord of MBP-deficient *shiverer/rag2* mice (Ehrlich et al., 2017). Similarly, iPSC-derived oligodendroglial cells from patients with primary progressive multiple sclerosis survived in *shiverer/rag2* mice and myelinated mouse axons of the corpus callosum 16 weeks after cell injection (Douvaras et al., 2014). These studies highlight the unique opportunity to test the function of patient-derived oligodendrocytes *in vivo* and to assess AD oligodendrocyte-driven mechanisms of disease development in chimeric AD mouse brains.

Future studies could involve the injection of multiple neural types into the brains at the same time, for instance to study how human neurons interact with human microglia, astrocytes or oligodendroglia in the AD brain. Added levels of complexity can be explored with this multi-lineage transplantation approach, which could also include co-injection of pathological proteins such as sarkosyl-insoluble tau isolated from AD patient brains to assess potential modulation of tau pathology and the spread of tau by human cells. Human-mouse chimeric models can also be used to study how human cells react to AD drugs in an *in vivo* setting. Species-specific differences in drug efficacy, metabolism, and toxicity have been widely described (Van Norman, 2020). This limitation demonstrates a real need for human-based systems, that human stem cell-based transplantation studies might be able to provide. In addition, the intracranial injection of human neural cells may, by itself, hold a therapeutic potential. Transplantation techniques could be further refined to slowly replace dying neurons with healthy neurons or to apply microglia or astrocytes as vehicles for delivery of beneficial molecules, as previously described for human hematopoietic stem and progenitor cells injected into the ventricle and brain parenchyma of mice (Capotondo et al., 2017). As seen here, cell transplantation offers a wide range of opportunities for exploring human-specific disease mechanisms *in vivo*. As the field progresses, it may even have the potential to support the development of therapeutic strategies for the benefit of patients.

## Author contributions

NI: Visualization, Writing – original draft, Writing – review & editing. PC: Conceptualization, Writing – original draft, Writing – review & editing. GH: Conceptualization, Funding acquisition, Resources, Supervision, Visualization, Writing – original draft, Writing – review & editing.
